# Outcomes of specialist discharge coordination and intermediate care schemes for patients who are homeless: analysis protocol for a population-based historical cohort

**DOI:** 10.1136/bmjopen-2017-019282

**Published:** 2017-12-14

**Authors:** Ruth M Blackburn, Andrew Hayward, Michelle Cornes, Martin McKee, Dan Lewer, Martin Whiteford, Dee Menezes, Serena Luchenski, Alistair Story, Spiros Denaxas, Michela Tinelli, Fatima B Wurie, Richard Byng, Michael C Clark, James Fuller, Mark Gabbay, Nigel Hewett, Alan Kilmister, Jill Manthorpe, Joanne Neale, Robert W Aldridge

**Affiliations:** 1 Institute of Health Informatics, University College London, London, UK; 2 Farr Institute of Health Informatics Research, University College London, London, UK; 3 Social Care Workforce Research Unit, King’s College London, London, UK; 4 Department of Health Services Research & Policy, London School of Hygiene and Tropical Medicine, London, London, UK; 5 Department of Health Services Research, University of Liverpool, Liverpool, UK; 6 University College London Hospitals, London, UK; 7 Personal Social Services Research Unit (PSSRU), London School of Economics and Political Science, London, UK; 8 Community and Primary Care Research Group, Plymouth University Peninsula Schools of Medicine and Dentistry, PLYMOUTH, UK; 9 Pathway Charity, London, UK; 10 National Addiction Centre, Institute of Psychiatry, Psychology and Neuroscience, King’s College London, London, UK

**Keywords:** homelessness, hospital discharge, intermediate care, medical respite

## Abstract

**Introduction:**

People who are homeless often experience poor hospital discharge arrangements, reflecting ongoing care and housing needs. Specialist integrated homeless health and care provision (SIHHC) schemes have been developed and implemented to facilitate the safe and timely discharge of homeless patients from hospital. Our study aims to investigate the health outcomes of patients who were homeless and seen by a selection of SIHHC services.

**Methods and analysis:**

Our study will employ a historical population-based cohort in England. We will examine health outcomes among three groups of adults: (1) homeless patients seen by specialist discharge schemes during their hospital admission; (2) homeless patients not seen by a specialist scheme and (3) admitted patients who live in deprived neighbourhoods and were not recorded as being homeless. Primary outcomes will be: time from discharge to next hospital inpatient admission; time from discharge to next accident and emergency attendance and 28-day emergency readmission. Outcome data will be generated through linkage to hospital admissions data (Hospital Episode Statistics) and mortality data for November 2013 to November 2016. Multivariable regression will be used to model the relationship between the study comparison groups and each of the outcomes.

**Ethics and dissemination:**

Approval has been obtained from the National Health Service (NHS) Confidentiality Advisory Group (reference 16/CAG/0021) to undertake this work using unconsented identifiable data. Health Research Authority Research Ethics approval (REC 16/EE/0018) has been obtained in addition to local research and development approvals for data collection at NHS sites. We will feedback the results of our study to our advisory group of people who have lived experience of homelessness and seek their suggestions on ways to improve or take this work further for their benefit. We will disseminate our findings to SIHHC schemes through a series of regional workshops.

**Strengths and limitations of this study**

- Our study will use data linkage to facilitate large-scale evaluation of care for homeless people; a group with significant health needs that is highly mobile and difficult to monitor.
- Data linkage will enable the health of homeless people admitted to hospital to be characterised and changes in their health evaluated with respect to access to specialist integrated homeless health and care schemes.
- Our analysis will be limited to individuals for whom deterministic linkage was possible and restricted to data collected in secondary care settings and mortality.

## Introduction

Homelessness is associated with high mortality,^1 2^ multiple morbidity and low uptake of preventative interventions.^3^ The most comprehensive assessment to date of the healthcare usage by homeless people in England was published by the Department of Health in March 2010.^4^ This report estimated that healthcare costs ascribed to homeless people were at least £85 million per year, which is approximately eight times greater—with threefold longer duration of hospital admission—than those of similarly aged adults.^4^

People who are homeless often experience poor hospital discharge arrangements, with the potential to further increase costs through increased rates of readmission. In 2013, the Department of Health launched the ‘Homeless Hospital Discharge Fund’ (HHDF)—a £10 million programme allocated to the voluntary sector to develop pilot projects to improve hospital discharge procedures for homeless patients through development of specialist integrated homeless health and care (SIHHC) schemes.^5^ This was partly a response to the finding that 70% of people who are homeless were being discharged from hospital back to the streets without having their housing or ongoing care needs properly addressed.^6^ In total, 52 discharge coordination and intermediate care ‘type’ SIHHC schemes were funded through the HHDF. According to an early evaluation report,^7^ the schemes fall into two broad categories:

- Housing-led schemes: These focus primarily on securing accommodation for people who are homeless on discharge from hospital. Some schemes provide short-term intermediate care follow-up in the community or in a hostel type setting.
- Clinically led schemes: These are usually general practitioner or nurse led and involve ‘in reach’ (hospital ward rounds) and discharge coordination with a focus on both health and housing. These schemes are often referred to as ‘pathway discharge teams’ in acknowledgement of the Pathway charity that pioneered this way of working.^8^

The clinically led approach has been evaluated in both observational^8^ and interventional^9^ study designs. These analyses found that the duration of hospital admission was not reduced, but quality of life was improved, street ­homelessness was reduced and patients felt better cared for. Evaluations of schemes using housing support workers tend to be smaller in scale, qualitative and not published in the academic literature.

This study explores the effectiveness of SIHHC as compared with standard care (ie, hospitals which do not have access to a specialist scheme to manage the discharge of patients who are homeless). Our evaluation will work with a sample of 17 SIHHC sites, most of which received funding through the HHDF. We aim to include 7 schemes that are clinically led and 10 that are housing led. Sites will be selected to represent different contexts and localities across England (eg, rural, city, inner London). Our research will be geographically more representative than previous analyses and the use of administrative records may offer a more realistic evaluation of the benefits of the intervention than measurements taken in an interventional study setting. This study contributes to a larger National Institute for Health Research (NIHR)-funded realist evaluation that seeks to understand ‘what works’ in the delivery of SIHHC. The full protocol for all related work packages is available from the NIHR website.^10^

### Methods and analysis

The study is a historical population-based cohort study. Eligible participants will be adults over 18 years of age with one or more hospital admissions between 1 November 2013 and 30 November 2016.

Individuals will enter the cohort at whichever is the latest of: 1 November 2013; their 18th birthday; hospital admission (‘index’ admission); SIHHC implementation date. Individuals will be followed up until the earliest of: 30 November 2016, their 100th birthday, death.

For each participant, the index admission will be identified. This is the first admission after the implementation of the SIHHC in the hospital concerned and thus the first at which they could potentially benefit from the scheme, regardless of whether or not they used it. The number of people who died during their index admission will be reported, but these individuals will be excluded and their data will not be included in the analysis beyond this point. Multiple admissions for the same individual less than 1 day apart, including those relating to a hospital transfer, will be assumed to be part of the same admission.^11^

### Ethics and information governance

**Figure 1:**
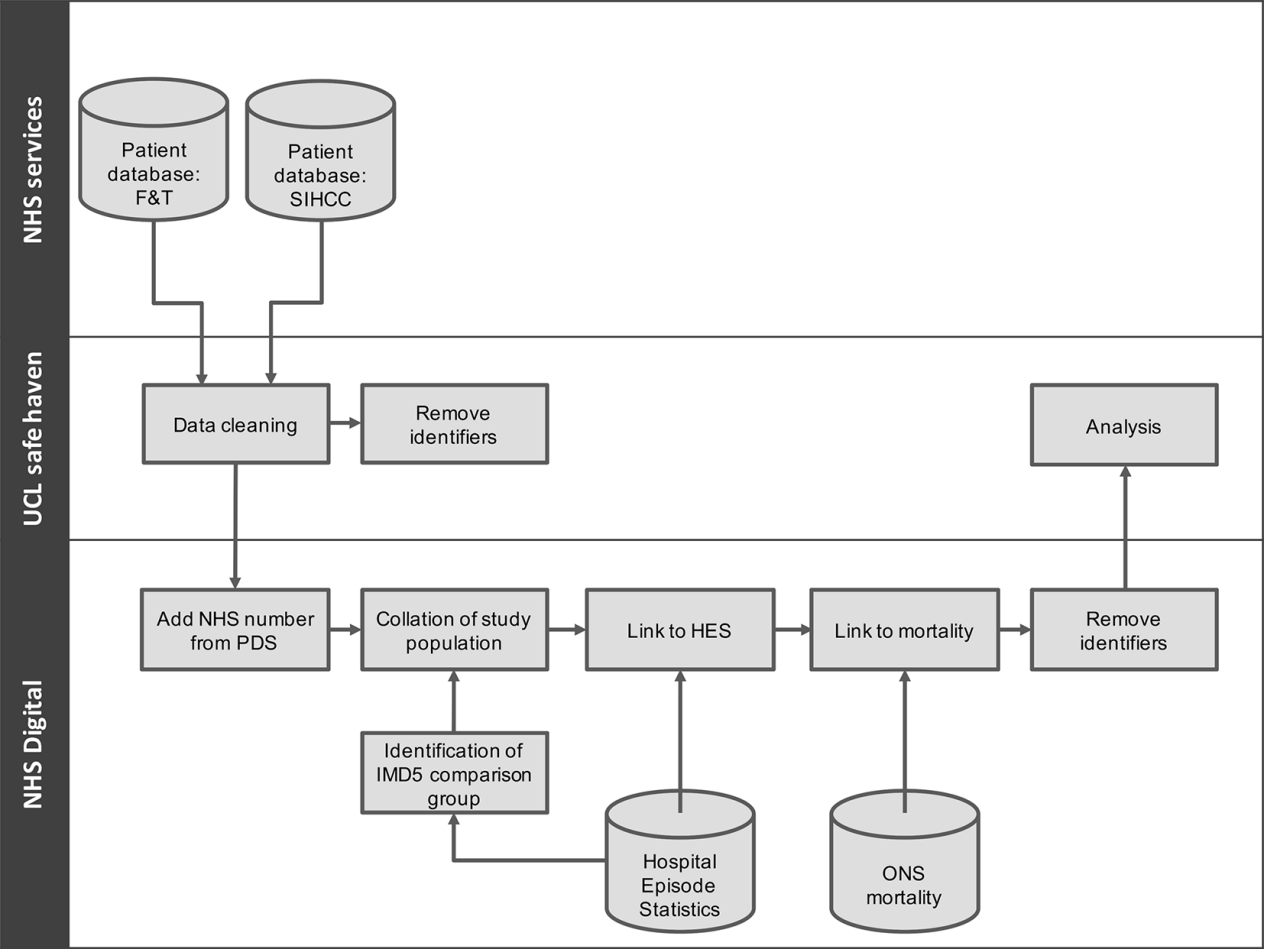
Study data flows. F&T, Find and Treat; HES, Hospital Episode Statistics; IMD, index of multiple deprivation; NHS, National Health Service; ONS, Office for National Statistics; PDS, Personal Demographics Service; SIHHC, specialist integrated homeless health and care; UCL, University College London.

Health Research Authority Research Ethics Committee approval has been sought and received (REC 16/EE/0018). In addition, local research and development approvals were set up prior to local data collection at each of the 17 SIHHC sites. To undertake this work, we require access to patient identifiable data without individual consent and have obtained approval (reference 16/Confidentiality Advisory Group (CAG)/0021) from the Secretary of State for Health through the CAG for this work. After data linkage (see Data collection, processing and linkage section), we will destroy all identifying data as shown in figure 1 and undertake all analyses using a deidentified dataset. All study data will be stored on the University College London (UCL) Data Safe Haven, which has been certified to the ISO27001:2013 information security standard and conforms to the National Health Service (NHS) Information Governance Toolkit.^12^

## Comparator groups

Three groups of individuals admitted to hospital will be included in the analysis. The first group comprises homeless individuals admitted to hospital at any one of 17 sites with a SIHHC scheme between 1 November 2013 and 30 November 2016. We have conducted a comprehensive audit of all SIHHC hospital sites as part of the wider NIHR evaluation. Using these data, we have determined the date on which SIHHC schemes were implemented.

The second group comprises individuals seen by a community homeless service in London (Find and Treat (F&T)) and subsequently admitted to a hospital that did not have a SIHHC scheme. For these non-SIHHC hospitals, a hypothetical implementation date will be chosen from within a randomly drawn decile of SIHHC implementation dates. F&T is a specialist outreach team that works alongside over 200 NHS and third sector front-line services to prevent or treat tuberculosis among homeless people. F&T is primarily based in London—an issue we will explore further in a sensitivity analysis by restricting the cohort to admissions in London alone. This group is subsequently referred to as F&T. We will look for hospital admissions during the study period for all individuals seen by F&T between 1 November 2013 and 30 November 2016.

The third group is a random sample of individuals equal in size to the F&T group and living in lower super output areas in England in the most deprived quintile, as measured by the index of multiple deprivation (IMD), who are recorded as having a fixed address. These individuals will come from hospitals offering SIHHC schemes with an admission during the study period but without being seen by the SIHHC scheme. This group will be used to confirm that the homeless people included in the study have worse outcomes than non-homeless people living in deprived areas. This group is subsequently referred to as IMD5.

### Datasets used in the analysis

We will draw on three sources of study participants. First, unconsented data collected at SIHHC sites (see ethics and data security section in relation to the approvals and information governance framework applied to carry out this work). This will include identifying demographic data (forename, surname, aliases, date of birth, sex, current or previous postcodes, nationality, ethnicity, NHS number—a unique 10-digit numeric identifier for patients in the healthcare system assigned at first encounter) for relevant hospital inpatients during the study period. Second, unconsented data from F&T, again including identifying demographic data. Third, hospital records from SIHHC sites for a random sample of anonymous patients from deprived areas will be generated, equal in size to the F&T group.

Data in respect of each group from Hospital Episode Statistics (HES), which includes dates, causes and length of admission and the Office for National Statistics’ mortality database, which includes dates and causes of death, will be linked. In this study, we will be unable to include data from primary care due to the lack of national dataset available for this purpose. As a result, we will be unable to examine any contribution to the health and care of the individuals from primary and community or social care, which is therefore an important limitation of the study.

### Data collection, processing and linkage

Data flows used in this analysis are described in figure 1. We will obtain demographic identifying variables from all individuals at SIHHC sites and F&T under our legal and ethical approvals. Where available, NHS number will be collected from the 17 SIHHC sites. NHS Digital (formally Health and Social Care Information Centre) will use the Personal Demographics Service (PDS) to identify and add NHS numbers to as many individual records as possible.

NHS Digital will undertake all data linkage to HES data using patient identifiers obtained from the SIHHC sites and F&T datasets (in combination with PDS tracing) and securely upload a deidentified copy of the data to the University College London Institute of Health Informatics’ Data Safe Haven—a robust infrastructure certified for processing and analysing identifiable data according to international and national information security standards (ISO/IEC 27001:2013 and NHS Information Governance Toolkit). The deterministic linkage process will be undertaken in three steps to ensure the three groups are mutually exclusive:

Step 1. HES linkage to data collected at the SIHHC sites.

Step 2. HES linkage to data collected from F&T community-based services, with admissions stratified into those sites with and without SIHHC schemes.

Step 3. Identifying people in the most deprived IMD quintile from HES at the SIHHC sites who were admitted after the implementation date at a given SIHHC site.

In the main analysis, individuals identified in more than one of the steps described above will be classified using the hierarchy 1>2>3, such that data on homeless patients who were admitted multiple times to a combination of hospitals with, and without, SIHHC schemes will be analysed according to admission to the SIHHC site only. Patients identified as both homeless (ie, SIHHC or F&T) as well as those in the non-homeless (IMD5) group will be assigned to the homeless category. Sensitivity analyses will examine the impact of excluding people who were identified in more than one group. We will obtain data on all individuals from HES in respect of admissions to any hospital in England from 1 January 2008 to 31 October 2013 to create a profile of their existing health conditions.

## Sample size

Our sample size is based on historical data from the two types of SIHHC scheme, suggesting an average of 92 patients per month across the 17 sites. We therefore estimate that each type will have data from approximately 2208 patients for the duration of the study.

From previous health service evaluations, we expect 0.7 hospital episodes per person-year for homeless individuals. A clinically important reduction in readmission rates would be 10%. To undertake a sample size calculation to determine the study size required to detect such a reduction in readmission rates, the following variables were defined:

μ_0=mean readmission rate in the baseline (F&T) group=0.7 episodes per person-year.

μ_1=mean readmission rate in the SIHHC group=0.6 episodes per person-year.

v=1.96=percentage point of the normal distribution corresponding to a 5% two-sided significance level.

u=0.84=one-sided percentage point of the normal distribution corresponding to (100%- type II error (FN/ (TP +FN))) at 80% power.

Using the following equation, we estimated minimum sample size (per group) required for comparison of two rates (readmissions per person-year):

(u+v)^2 * (μ_1 + μ_0) / (μ_1 – μ_0)^2

(0.84+1.96) ^2 *(0.7+0.6) / (0.7–0.6) ^2

=1019 person-years per comparator group

Adjustment for confounders (age, sex, ethnicity, calendar quarter of admission and existing comorbidities) as far as is possible within the analysis, results in a doubling of sample size (additional 10% per confounder). Therefore, 2038 person-years will be required per group. Assuming that the SIHHC sites see on average 90 patients each month during the study period (which for the purposes of the initial calculation we assumed to be November 2013–November 2015 as many schemes may have been shut down before this time period and in some we may collect data at an earlier point), we estimate that this translates to a total 2160 individuals at each of these two types of scheme. Given that the average follow-up period is likely to exceed 1 year, we anticipate that the study will be powered to detect a 10% difference in readmission rates between SIHHC and non-SIHHC sites, for both the clinically led and the housing-led SIHHC schemes.

## Outcomes

A series of primary and secondary outcomes will be included to ensure our analysis is both consistent with outcomes used in previous published analyses and includes outcomes that previous studies were not powered to collect. We have chosen a large number of secondary outcomes that collectively reflect the priorities of health policy-makers and individuals attending our patient engagement workshops. Full definitions of each primary and secondary outcome are provided in tables 1 and 2. In summary they are:

**Table 1:** Definition and methodological approach for primary outcomes

Primary outcome	Definition	Approach
Time from discharge to next hospital inpatient admission (any cause)	Binary indicator for readmission (yes/no). Time to event defined as index admission discharge date until the earliest of: readmission end of follow-up	Cox proportional hazards model
Time from discharge to next A&E attendance	Binary indicator for subsequent A&E attendance (yes/no). Time to event defined as index admission discharge date until the earliest of: next A&E attendance end of follow-up	
28-day emergency readmission	Binary indicator for emergency readmission (yes/no) recorded within 28 days of the index admission discharge date. Emergency admissions are defined as those where the admission method is 11, 12 or 13.	Logistic regression

A&E, accident and emergency.

**Table 2:** Definition and methodological approach for secondary outcomes

Secondary outcome	Definition	Approach
Time from admission to death	Binary indicator for death (yes/no). Time to event defined as index admission date until the earliest of: death end of follow up Deaths will primarily be identified through linkage to ONS deaths registration data. However, a supplementary analysis will use HES data (where the method of discharge field is coded as ‘dead’ (4)) in addition to ONS deaths data. This is because HES records may better ascertain information on recent deaths where there is a delay in death registration (eg, because a coroner’s report is required).	Cox proportional hazards model
Duration of index hospital admission	(Date of discharge)—(date of admission)	Poisson/Zero-inflated Poisson model
Time from admission to avoidable deaths	Binary indicator for avoidable death (yes/no). Time to event defined as index admission date until the earliest of: avoidable death^14^ end of follow up Supplementary analyses will investigate amenable and preventable deaths (see online supplementary file 1), which are a subset of avoidable deaths.	Cox proportional hazards model
Time from discharge to ACS condition admission	Binary indicator for admission with ACS (yes/no). Time to event defined as index discharge date until the earliest of: ACS condition admission end of follow up ACS admissions are flagged within HES 14	
Time from discharge to next elective admission	Binary indicator for elective readmission (yes/no). Time to event defined as index discharge date until the earliest of: elective readmission end of follow up Elective admissions are those where the admission method is 11, 12 or 13 in addition, a sensitivity analysis investigating planned admissions only (admission method 13) will be undertaken.^14^	
Overall readmission rates	Number of readmissions divided by the total time under follow up between admissions (ie, where the patient was not already hospitalised). Calculated as (number of admissions occurring in the time from index discharge date to the earliest of death or November 2016) divided by (number of days from index discharge date to the earliest of death or November 2016 minus the number of days in the same time period where the individual was admitted to hospital).	Poisson model with the log of follow-up time as an offset
Unscheduled readmission rates	As for overall readmission rates (above) but excluding (from the numerator only) admissions where the admission method was elective (ie, 11–13).	Poisson model with the log of follow-up time as an offset
All-cause mortality expressed as a standardised mortality ratio	Deaths will primarily be identified through linkage to ONS deaths registration data, but also through HES (where the method of discharge field is coded as ‘dead’ (4)) as the latter method may better ascertain information on recent deaths where there is a delay in death registration (eg, because a coroner’s report is required).	Calculation of SMR using Office of National Statistics death data by age and gender.
ICD-10 chapter specific SMR	As for all-cause mortality (above), but examining deaths by ICD-10 chapter for primary cause of death	
In-patient costs using HRG	Each entry will be assigned a unit cost based on its HRG. A total cost for each patient calculated as the sum of costs across all entries during the period.	A discounting rate of 3.5% will be applied and GLM modelling willbe undertaken with Gamma specification.

ACS, ambulatory care sensitive; GLM, generalised linear model; HES, Hospital Episode Statistics; HRG. Health Resource Group; ICD, International Classification of Diseases 10th Revision; ONS, Office for National Statistics; SMR, standardised mortality ratio.

10.1136/bmjopen-2017-019282.supp1 Supplementary file 1

### Primary outcomes

- Time from discharge to next hospital inpatient readmission (any cause)
- Time from discharge to next accident and emergency attendance
- 28-day emergency readmission

### Secondary outcomes

- Time from admission to death due to any cause
- Duration of inpatient admission
- Time from admission to mortality from causes amenable to healthcare^13^
- Time from discharge to admission with ambulatory care sensitive (ACS) conditions
- Time from discharge to next planned admission
- Overall readmission rates
- Unscheduled readmission rates
- All-cause mortality using a standardised mortality ratio (SMR)
- International Classification of Diseases 10th Revision chapter-specific SMR
- In-patient costs using Health Resource Group

## Analysis plan

**Figure 2:**
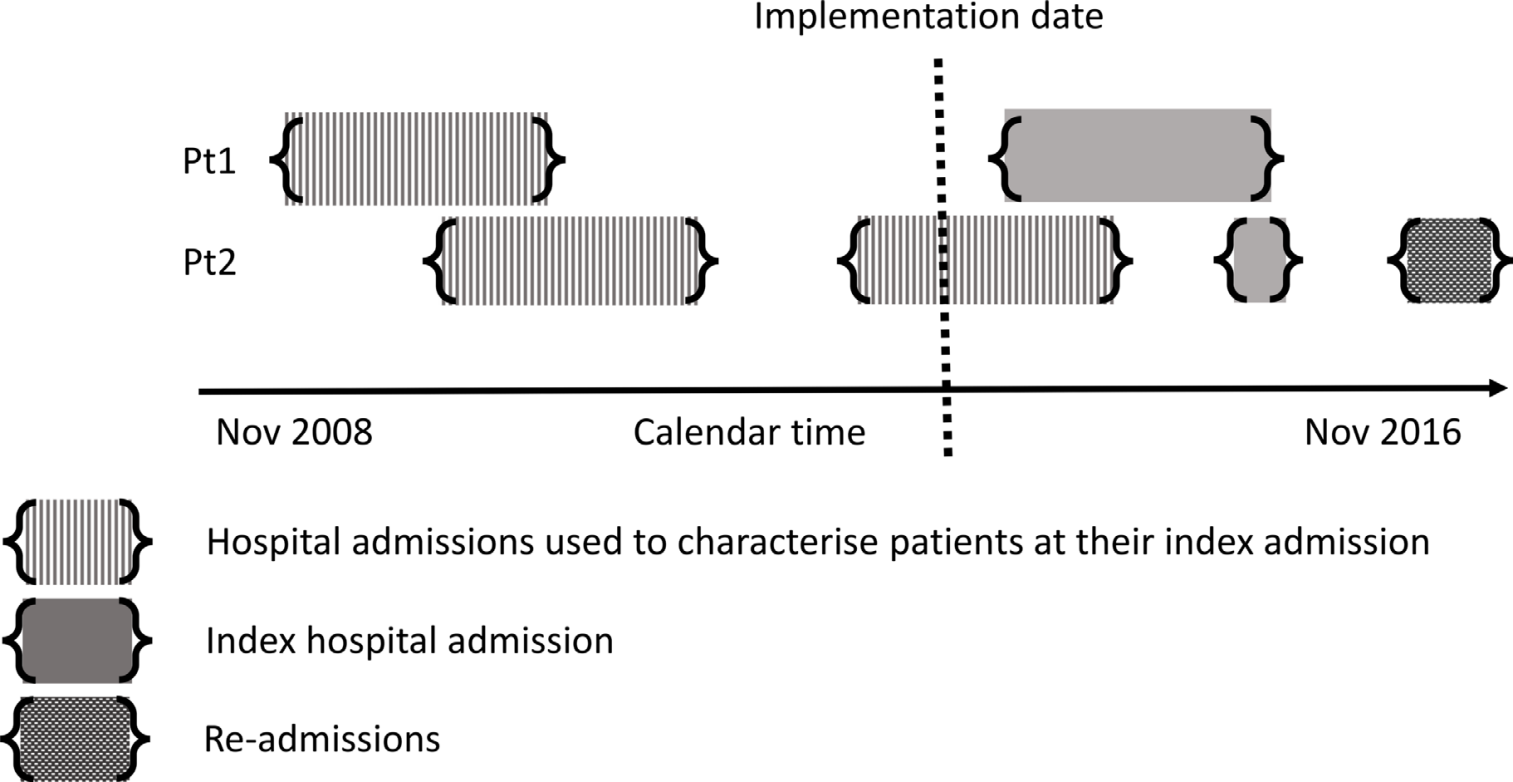
Schematic outlining hypothetical patients. Data on the characteristics of patients before their index admission will be collated from admissions occurring before the implementation of SIHHC schemes at a given site (or for comparator groups a randomly selected date within the range of implementation dates for SIHHC sites). SIHHC, specialist integrated homeless health and care provision.

We will undertake the analysis in two phases. In the first phase, we will analyse baseline characteristics (see table 3) of all participants to describe the characteristics of each of the study groups at or before the index admission (see figure 2). With the exception of ethnicity, all baseline characteristics are anticipated to be fully observed (chronic disease is presumed to be absent unless recorded). Missing values of ethnicity will be analysed grouped as ‘not recorded’.

**Table 3:** Patient characteristics in the time prior to the index admission will be collated as baseline measurements

Variable	Description
Age	(In years) at a given time point will be estimated as ((date of admission – month and year of birth)/365.25) for the index admission
Sex	As recorded at the index admission*
Ethnicity	As recorded at the index admission*
ICD-10 chronic disease conditions	Obtained from all admissions at or before the index admission, subdivided into categories of: 1. mental health/behavioural 2. cancer/blood disorders 3. chronic infections 4. respiratory 5. metabolic/endocrine/nutritional 6. renal/genitourinary 7. musculoskeletal/dermatological 8. neurological 9. cardiovascular
Admitting diagnosis	Reason for index hospital admission classified according to HRG, which describes case-mix according to the chapter and subchapter of the reason for admission and associated procedures.

*Missing information in the index admission record will be completed (where possible) with the modal value from other records for the same individual.

HRG, Health Resource Group; ICD-10, International Classification of Diseases 10th Revision.

We will summarise each of the primary and secondary outcomes by comparison group and explore the geographical spread of our 17 sites to explore their representativeness. This work will enable us to confirm the suitability of the proposed statistical methods and analysis protocol proposed. In addition, we will use life tables to estimate the life expectancy of homeless people included within the study, and we will examine overall rates of avoidable deaths and ACS as these have all been poorly estimated within the literature prior to this study.

In the second phase, we will identify evidence of differences in the baseline characteristics including age, sex, chronic disease and reason for hospital admission at the time of their index admission. We will estimate the crude association between each of the primary and secondary outcomes and the study population groups. The baseline comparator group will be F&T. We will then re-estimate the association between each of the outcomes and the study population group after adjusting for characteristics at the time of admission: age, sex, chronic disease and reason for hospital admission. Finally, we will undertake supplementary subgroup analyses to evaluate evidence of a difference in the outcomes of people admitted to clinically led versus housing-led schemes.

An appropriate statistical model (selected on the basis of meeting assumptions such as proportional hazards for Cox regression) will be used to analyse the relationship between the study comparison group and each of the outcomes outlined in Tables 1,2. Crude models will be fitted prior to adjustment for ‘baseline’ measurements at or before the index admission. We will write the analysis in accordance with the REporting of studies Conducted using Observational Routinely-collected Data statement.^14^

## Sensitivity analyses

There are several challenges when examining differences in the outcomes for users of SIHHC schemes. First, there are likely to be differences in the underlying health risks of the groups and accessibility of services. Second, clinically led schemes may be more likely to collect NHS numbers, potentially improving (and biassing) the accuracy of linkage for this group compared with others. Third, there may be biases between the groups in their propensity to use health services in the immediate period after the study. Those using clinically led schemes may be more likely to return to these services again and be captured more often within HES data than the schemes led by housing support workers. Fourth, it is possible that individuals seen by SIHHC schemes are the most unwell and therefore have poor outcomes regardless of services. Conversely, it is possible (although we think unlikely) that individuals who are homeless compared with those who are housed end up being admitted earlier and have less mortality and morbidity. Our range of primary and secondary outcomes and sensitivity analyses will help us explore these issues further. While we will provide point estimate of effectiveness, more importantly, we believe that our study will produce a plausible range of outcomes for the carefully chosen comparator groups (using confidence and uncertainty intervals).

We will examine these plausible ranges further by conducting the following sensitivity analyses. First, we will repeat analysis only for those individuals for whom it has been possible to generate confounding variables that can be used for adjustment within the statistical models. Second, we will designate a wash out period of 3 months after the index admission date where outcome data for this period are excluded to reduce bias of healthcare usage by the initial SIHHC scheme. Third, we investigate the impact of restricting follow-up time to the 6 and 12-month time periods following implementation dates as many of the SIHHC schemes were only operational for 6 to 12 months. Fourth, we will identify deaths using HES data (Method of discharge field, code 4) in addition to deaths identified using Office for National Statistics mortality data.^15^ Fifth, we will conduct separate analyses according to the type of index admission (elective or emergency). Sixth, we will investigate only rates of planned elective readmissions (admission method 13). Seventh, we will restrict our analysis to admissions in London to explore any bias in a comparison with F&T which undertakes the majority of its screening in community settings in London.

### Dissemination and impact

To help ensure impact from the work, we have engaged with different groups throughout the design and conduct of the study to ensure relevance to their worlds and prepare a pathway for impact and will continue to do this through to the end of the project. In designing this study, we held a workshop with people who had lived experience of homelessness to understand their views on the consent process, data linkage and analysis. At the end of the study, we will reconvene a similar group to feedback the results of our study and seek suggestions on ways to take this work forward. We will also disseminate our findings to SIHHC schemes through a series of regional workshops.

## Supplementary Material

Reviewer comments
