# Supplementary material for: Outcomes of specialist discharge coordination and intermediate care schemes for patients who are homeless: analysis protocol for a population-based historical cohort
Source: BMJ Open. 2017 Dec 14;7(12):e019282. doi: 10.1136/bmjopen-2017-019282 (PMC5736042; doi:10.1136/bmjopen-2017-019282)
Supplement: Supplementary file 1 [file bmjopen-2017-019282supp001.pdf]

Supplementary Appendix

Office of National Statistics Definition of Avoidable Mortality (2016):

Amenable mortality

A death is amenable if, in the light of medical knowledge and technology at the time of death, all or most deaths from that cause (subject to age limits if appropriate) could be avoided through good quality healthcare.

Preventable mortality

A death is preventable if, in the light of understanding of the determinants of health at the time of death, all or most deaths from that cause (subject to age limits if appropriate) could be avoided by public health interventions in the broadest sense.

Avoidable mortality

Avoidable deaths are all those defined as preventable, amenable, or both, where each death is counted only once. Where a cause of death falls within both the preventable and amenable definition, all deaths from that cause are counted in both categories when they are presented separately.

Table S1 – developed by ONS – outlines specific ICD-10 codes relating to Avoidable (all in table), Preventable (right-hand column) and Amenable (second-to last column) mortality.

Condition group and cause	ICD-10 codes	Age	Amenable	Preventable
**Infections**				
Tuberculosis	A15-A19, B90	0-74	•	•
Selected invasive bacterial and protozoal infections	A38-A41, A46, A48.1, B50-B54, G00, G03, J02, L03	0-74	•	
Hepatitis C	B17.1, B18.2	0-74	•	•
HIV/AIDS	B20-B24	All	•	•
**Neoplasms**				
Malignant neoplasm of lip, oral cavity and pharynx	C00-C14	0-74		•
Malignant neoplasm of oesophagus	C15	0-74		•
Malignant neoplasm of stomach	C16	0-74		•
Malignant neoplasm of colon and rectum	C18-C21	0-74	•	•
Malignant neoplasm of liver	C22	0-74		•
Malignant neoplasm of trachea, bronchus and lung	C33-C34	0-74		•
Malignant melanoma of skin	C43	0-74	•	•
Mesothelioma	C45	0-74		•
Malignant neoplasm of breast	C50	0-74	•	•
Malignant neoplasm of cervix uteri	C53	0-74	•	•
Malignant neoplasm of bladder	C67	0-74	•	
Malignant neoplasm of thyroid gland	C73	0-74	•	
Hodgkin's disease	C81	0-74	•	
Leukaemia	C91, C92.0	0-44	•	
Benign neoplasms	D10-D36	0-74	•	
**Nutritional, endocrine and metabolic**				
Diabetes mellitus	E10-E14	0-49	•	•
**Drug use disorders**				
Alcohol related diseases, excluding external causes	F10, G31.2, G62.1, I42.6, K29.2, K70, K73, K74 (excl. K74.3-K74.5), K86.0	0-74		•
Illicit drug use disorders	F11-F16, F18-F19	0-74		•
**Neurological disorders**				
Epilepsy and status epilepticus	G40-G41	0-74	•	

Condition group and cause	ICD-10 codes	Age	Amenable	Preventable
**Cardiovascular diseases**				
Rheumatic and other valvular heart disease	I01-I09	0-74	•	
Hypertensive diseases	I10-I15	0-74	•	
Ischaemic heart disease	I20-I25	0-74	•	•
DVT with pulmonary embolism	I26, I80.1-I80.3, I80.9, I82.9	0-74		•
Cerebrovascular diseases	I60-I69	0-74	•	
Aortic aneurysm and dissection	I71	0-74		•
**Respiratory diseases**				
Influenza (including swine flu)	J09-J11	0-74	•	•
Pneumonia	J12-J18	0-74	•	
Chronic obstructive pulmonary disorder	J40-J44	0-74		•
Asthma	J45-J46	0-74	•	
**Digestive disorders**				
Gastric and duodenal ulcer	K25-K28	0-74	•	
Acute abdomen, appendicitis, intestinal obstruction, cholecystitis/lithiasis, pancreatitis, hernia	K35-K38, K40-K46, K80-K83, K85, K86.1-K86.9, K91.5	0-74	•	
**Genitourinary disorders**				
Nephritis and nephrosis	N00-N07, N17-N19, N25-N27	0-74	•	
Obstructive uropathy and prostatic hyperplasia	N13, N20-N21, N35, N40, N99.1	0-74	•	
**Maternal and infant**				
Complications of perinatal period	P00-P96, A33	All	•	
Congenital malformations, deformations and chromosomal anomalies	Q00-Q99	0-74	•	
**Unintentional injuries**				
Transport Accidents	V01-V99	All		•
Accidental Injury	W00-X59	All		•
**Intentional injuries**				
Suicide and self inflicted injuries	X60-X84, Y10-Y34	All		•
Homicide/Assault	X85-Y09, U50.9	All		•
Misadventures to patients during surgical and medical care	Y60-Y69, Y83-Y84	All	•	•
