# Supplementary material for: Outcomes of specialist discharge coordination and intermediate care schemes for patients who are homeless: analysis protocol for a population-based historical cohort
Source: BMJ Open. 2017 Dec 14;7(12):e019282. doi: 10.1136/bmjopen-2017-019282 (PMC5736042; doi:10.1136/bmjopen-2017-019282)
Supplement: Reviewer comments [file bmjopen-2017-019282.reviewer_comments.pdf]

PEER REVIEW HISTORY

BMJ Open publishes all reviews undertaken for accepted manuscripts. Reviewers are asked to complete a checklist review form (**http://bmjopen.bmj.com/site/about/resources/checklist.pdf**) and are provided with free text boxes to elaborate on their assessment. These free text comments are reproduced below.

ARTICLE DETAILS

**TITLE (PROVISIONAL)**	Outcomes of Specialist Discharge Coordination and Intermediate Care Schemes for Patients who are Homeless: Analysis protocol for a population-based historical cohort
**AUTHORS**	Blackburn, Ruth; Hayward, Andrew; Cornes, Michelle; McKee, Martin; Lewer, Dan; Whiteford, Martin; Menezes, Dee; Luchenski, Serena; Story, Alistair; Denaxas, S; Tinelli, M; Wurie, Fatima; Byng, Richard; Clark, Michael; Fuller, James; Gabbay, Mark; Hewett, Nigel; Kilmister, Alan; Manthorpe, Jill; Neale, J; Aldridge, Robert

This article was not externally reviewed at BMJ Open. Protocols that have been independently assessed prior to submission to BMJ Open are usually fast-tracked to publication on the grounds that further substantial changes will not be possible. This independent assessment will usually be external, independent review for both a substantial grant award from a non-commercial or government funder and ethics approval.
